# Maternal intake of grape seed procyanidins during lactation induces insulin resistance and an adiponectin resistance-like phenotype in rat offspring

**DOI:** 10.1038/s41598-017-12597-9

**Published:** 2017-10-03

**Authors:** Antoni Caimari, Roger Mariné-Casadó, Noemí Boqué, Anna Crescenti, Lluís Arola, Josep Maria del Bas

**Affiliations:** 1 Technological Unit of Nutrition and Health. EURECAT-Technology Centre of Catalonia, Reus, Spain; 2 Nutrition and Health Research Group, EURECAT-Technology Centre of Catalonia, Reus, Spain; 3 Nutrigenomics Research Group, Department of Biochemistry and Biotechnology, Universitat Rovira i Virgili, Tarragona, Spain

## Abstract

Previously, we demonstrated that a grape seed procyanidin extract (GSPE) supplementation in pregnant and lactating rats exerted both healthy and deleterious programming effects on their offspring. Here, we evaluated whether the administration of GSPE during lactation (100 mg.kg^−1^.day^−1^) in rats elicited beneficial effects in their normoweight (STD-GSPE group) and cafeteria-fed obese (CAF-GSPE group) adult male offspring. STD-GSPE and CAF-GSPE offspring showed increased energy expenditure and circulating total and high-molecular-weight adiponectin. However, these rats showed hyperinsulinemia, decreased insulin sensitivity, increased insulin resistance, down-regulated mRNA levels of adiponectin receptors in inguinal white adipose tissue (*Adipor1 and Adipor2*) and soleus muscle (*Adipor2*), and decreased levels of phosphorylated AMPK, the downstream post-receptor target of adiponectin, in the soleus muscle. These deleterious effects could be related to an increased lipid transfer to the pups through the milk, since GSPE-supplemented dams displayed decreased fat content and increased expression of lipogenic genes in their mammary glands, in addition to increased circulating total adiponectin and non-esterified free fatty acids. In conclusion, maternal intake of GSPE during lactation induced insulin resistance and an adiponectin resistance-like phenotype in their normoweight and obese offspring. These findings raise concerns about the possibility of using GSPE as a nutraceutical supplement during this period.

## Introduction

Metabolic syndrome (MetS), which can be defined as a cluster of interconnected risk factors including insulin resistance, obesity, dyslipidemia and hypertension, is associated with an increased risk of cardiovascular disease (CVD) and type-2 diabetes, which are the two primary causes of mortality worldwide^1^. Thus, the World Health Organization estimated that the leading cause of non-communicable disease deaths in 2012 was CVD (17.5 million deaths), while diabetes caused another 1.5 million deaths. Even more alarming is the clear relationship demonstrated between the nutritional and metabolic disturbances during prenatal and early postnatal periods and the subsequent development of diseases in adult life^2–4^. Concomitantly, there is growing evidence that children of mothers with gestational diabetes are more prone to develop type-2 diabetes later in life^4^, and obesity before and during early pregnancy increases the risk of obesity and metabolic and cardiovascular disorders in adulthood^3^. All of these concerns highlight the need for deeper insight into the disease mechanisms and innovative nutritional strategies for combating these pathologies.

Many studies showed that polyphenols, bioactive food compounds present in fruits and vegetables, exert many beneficial effects related to the prevention or amelioration of CVD and different features of MetS^5,6^. Previous studies including our research demonstrated the effectiveness of a grape seed procyanidin extract (GSPE) against insulin resistance^7^, dyslipidemia^8^, increased adiposity^9^, hypertension^10^ and inflammation^11^. Additionally, different studies have demonstrated that maternal consumption of polyphenols during prenatal and early postnatal periods can positively affect the health of the offspring^12–18^. Regarding GSPE, we showed that procyanidins could target the placenta and fetus^19^ and that 30-day-old male offspring of dams fed a high-fat diet (HFD) and supplemented with this extract during pregnancy and lactation showed a better inflammatory profile in the blood and the epididymal white adipose tissue (EWAT)^17^. Furthermore, maternal intake of GSPE also exerted beneficial effects on the male normoweight offspring in adulthood, promoting whole-body fat oxidation probably via the up-regulation of phosphorylated AMP-activated protein kinase (p-AMPK) in skeletal muscle^18^. However, the effect of administration of GSPE during pregnancy and/or lactation in ameliorating MetS in the adult offspring has not yet been tested.

By contrast, we also demonstrated that GSPE supplementation in rats during pregnancy and lactation increased the body fat content of the HFD-fed male offspring in youth^17^ and disrupted the reverse cholesterol transport and increased atherogenic risk indexes in the adult male normoweight offspring^20^. Moreover, we also showed that dams receiving GSPE during these periods displayed increased circulating levels of insulin^18^. In addition, other studies showed that polyphenols or polyphenol-enriched foods^21–24^ exerted deleterious effects concerning fertility, pregnancy and foetal development, kidney structure and bone mineralization when were administered mainly during pregnancy. Thus, we hypothesized that the intake of GSPE exclusively during lactation, a less fragile and sensitive period than gestation, would be a more suitable approach for obtaining the beneficial metabolic programming effects associated with polyphenol consumption.

The main aim of the present study was to evaluate, in lactating rats, the effect of maternal intake of moderate doses of GSPE (100 mg.kg^−1^.day^−1^) in attenuating obesity and/or its related metabolic disorders in young adult offspring fed a cafeteria diet (CAF). This animal model resembles the MetS that occurs in humans^25^. Furthermore, the effects of GSPE on dams and their offspring fed a standard chow diet (STD) were also studied.

## Methods

### Procyanidin extract

GSPE was obtained from white grape seeds and was kindly provided by Les Dérives Résiniques et Terpéniques (Dax, France). According to the supplier, the extract contained a 75% of procyanidins, analyzed by the Porter method, and its phenolic profile was as follows: monomers of flavan-3-ols (15.98%), dimers (13.05%), trimers (12.22%), tetramers (9.97%) and oligomers (5–13 units; 23.77%) of procyanidins. A subsequent HPLC-MS/MS analysis of the phenolic acids and flavan-3-ols present in the GSPE extract revealed that this extract mainly contained gallic acid (3.11%), protocatechuic acid (0.13%), epicatechin (9.34%), epicatechin gallate (2.12%), catechin (12.13%), procyanidin dimers B1 (8.88%), B2 (3.32%) and B3 (4.61%), dimer gallate (0.89%) and trimeric procyanidins (0.49%)^26^. The gross composition of the GSPE extract was also analyzed by a reference analytical laboratory (Agrolab Ibérica S.L.U., Tarragona, Spain). The extract contained 18.95% of sugars (quantified as glucose), 6.82% of humidity, 0.29% of fat and 0.11% of ash. The amounts of protein and dietary fiber were below the limit of detection (LOD), which was <2% and <1% for protein and fiber, respectively.

### Animals

The Animal Ethics Committee of the University Rovira i Virgili (Tarragona, Spain) approved all of the procedures. The experimental protocol followed the ‘Principles of laboratory animal care’, and was carried out in accordance to the European Communities Council Directive (86/609/EEC). All animals were housed at 22 °C with a light/dark cycle of 12 hours (lights on at 09:00 am) and were given free access to food and water.

Eighteen 11-week-old female virgin Wistar rats (Harlan Laboratories, Barcelona, Spain) were submitted to the breeding protocol that was previously described^18^. After adjustment of the litter size to ten pups per dam, the nursing rats were orally supplemented from day 1 to day 21 of lactation, at 09:00 am, with either low-fat condensed milk (VEH group) or 100 mg.kg^−1^.day^−1^ of GSPE dissolved in low-fat condensed milk (GSPE group). Considering the dam’s weight as 280 g, the dose of GSPE used was equivalent to the daily consumption of 1237 mg of GSPE for a 60-kg human^27^, a dosage that can be considered acceptable in a context of a nutraceutical supplementation. Taking into account that the extract approximately contains 75% of procyanidins, the extrapolated amount of flavan-3-ols administered (927.8 mg) is slightly lower than the estimated Spanish dietary intake of total polyphenols (1200 mg/day/person)^28^. Both groups of dams were fed with a STD (3.1 kcal/g; Teklad Global 18% Protein Rodent Diet 2018, Harlan, Barcelona, Spain). The body weight was recorded every 2 days, and the food intake was documented every 3–4 days. On day 21 of lactation, the dams were sacrificed under anesthesia (pentobarbital sodium, 60 mg.kg^−1^) after 6 h of fasting.

On postnatal day 21, male pups (at least one pup from each litter) of both groups of dams were randomly selected, weighted, single-caged and fed with either the STD or a CAF diet until the age of 90 days. This design resulted in four experimental groups (n = 10–13): STD-veh, STD-GSPE, CAF-veh and CAF-GSPE. The nutrient composition of CAF used herein as well as the caloric breakdown were recently described by Cigarroa *et al*.^29^. Body weight was recorded twice a week, and food intake was documented every 10 days. At the age of 90 days, the animals were sacrificed after 6 hours of fasting as described above.

In both studies, blood was collected by cardiac puncture, and plasma was obtained by centrifugation and stored at −20 °C until analysis. The tissues were rapidly removed after death, weighed, frozen in liquid nitrogen and stored at −80 °C until analysis.

### Adiposity

The adiposity index was computed as previously described^18^.

### Body composition analyses

Lean and fat mass measurements (in grams) were performed without anesthesia in both dams and in their STD-fed and CAF-fed offspring the last day of the study (at 09:00 am) using an EchoMRI-700™ device (Echo Medical Systems, L.L.C., Houston, USA). The measurements were performed in triplicate. Data are expressed in absolute values as well as in relative values (as a percentage of body weight).

### Plasma analyses

The circulating levels of total cholesterol, triglycerides, phospholipids, glucose, non-esterified free fatty acids (NEFAs), insulin, leptin, monocyte chemoattractant protein-1 (MCP-1) and total adiponectin at the end point were carried out using commercial kits as previously described^18,30^. The plasma levels of high-molecular-weight adiponectin (HMW-adiponectin) were quantified using a mouse/rat ELISA kit (Shibayagi Co., Ltd., Shibukawa, Japan).

### Oral glucose tolerance test (OGTT)

On postnatal day 80, the STD-veh, STD-GSPE, CAF-veh and CAF-GSPE groups were submitted to an OGTT as previously described^18^.

### HOMA-IR and R-QUICKI analyses

The homeostasis model assessment-estimated insulin resistance (HOMA-IR) and the revised quantitative insulin sensitivity check index (R-QUICKI) were calculated as previously described^13,30^.

### Indirect calorimetry and activity measurements

Indirect calorimetry and activity analyses were performed in dams (at day 19 of lactation) and their offspring (at day 83–84 of life) using the Oxylet Pro^TM^ System (PANLAB, Cornellà, Spain). The dams received the corresponding treatment at 09:00 am. Four hours after (at 01.00 pm) the nursing rats were separated from their pups and were transferred from their cages to an acrylic box with free access to water but no access to food. After an initial acclimatisation period of 1 hour, the indirect calorimetric analyses were carried out only for 3 hours (from 02:00 pm to 5:00 pm) to minimize the time of separation between them and their pups. In the offspring, these measurements were performed with free access to water, in *ad libitum* conditions and during 20 hours (from 12.00 pm to 08.00 am) after allowed to become acclimated to the acrylic cages for 3.5 hours (from 08:30 am to 12:00 pm). The respiratory quotient (RQ), Energy Expenditure (EE) and the rates of fat and carbohydrate oxidation were calculated as previously described^18^. Locomotor activity was measured by continuous recording of spontaneous activity through extensiometric weight transducers placed below the home cage, and the number of rearings was monitored by 2-dimensional infrared frame elements, using the software program Metabolism 2.1.02 (PANLAB, Cornellà, Spain). In both experiments, temperature was controlled at 22 °C.

### Gene expression analyses

Extraction of RNA, cDNA synthesis and Real time-quantitative-PCR from liver, white adipose tissue, mammary gland, and soleus muscle samples were carried out as previously described^18^, using the oligonucleotides included in Table 1. Each PCR was performed at least in duplicate, and hypoxanthine guanine phosphoribosyl transferase (*Hprt*), peptidylprolyl isomerase (*Ppia*), transferrin receptor *(Tfrc*) and actin beta (*β-actin*) were used as reference genes.

**Table 1:** Nucleotide sequences of primers used for real-time quantitative PCR.

Gene	Forward primer (5′ to 3′)	Reverse primer (5′ to 3′)
*Acc1*	TGCAGGTATCCCCACTCTTC	TTCTGATTCCCTTCCCTCCT
*Adipoq*	GTTCCAGGACTCAGGATGCT	CGTCTCCCTTCTCTCCCTTC
*Adipor1*	TCTCCATCGTCTGTGTCCTG	AATCCGAGCAGCATAAAGGC
*Adipor2*	AGCCATTCTCTGCCTTTCCT	ACATGTCCCACTGAGAGACG
*Atgl*	CACTTTAGCTCCAAGGATGA	TGGTTCAGTAGGCCATTCCT
*β-actin*	TACAGCTTCACCACCACAGC	TCTCCAGGGAGGAAGAGGAT
*Cd36*	GTCCTGGCTGTGTTTGGA	GCTCAAAGATGGCTCCATTG
*Cpt1α*	GCTCGCACATTACAAGGACAT	TGGACACCACATAGAGGCAG
*Cpt1β*	GCAAACTGGACCGAGAAGAG	CCTTGAAGAAGCGACCTTTG
*Dgat1*	CAGACAGCGGTTTCAGCAAT	AGGGGTCCTTCAGAAACAGAG
*DsbA-L*	GCTTCACGTTCGCTTCTCTC	GCCGCAACTTCAGCTTGATA
*Ero1-Lα*	TGTCAAACCCTGCCATTCTG	TCCACATACTCAGCATCGGG
*Fas*	CGGCGAGTCTATGCCACTAT	ACACAGGGACCGAGTAATGC
*Gpat*	CAGCGTGATTGCTACCTGAA	CTCTCCGTCCTGGTGAGAAG
*Had*	ATCGTGAACCGTCTCTTGGT	AGGACTGGGCTGAAATAAGG
*Hprt*	TCCCAGCGTCGTGATTAGTGA	CCTTCATGACATCTCGAGCAAG
*Hsl*	TCACGCTACATAAAGGCTGCT	CCACCCGTAAAGAGGGAACT
*Pparα*	GTGGCTGCTATAATTTGCTGTG	AGCTTCGGGAAGAGAAAGGTAT
*Ppia*	CCAAACACAAATGGTTCCCAGT	ATTCCTGGACCCAAAACGCT
*Tfrc*	ATCATCAAGCAGCTGAGCCAG	CTCGCCAGACTTTGCTGAATTT

Primer pairs for PCR were designed using Primer3 software, and the sequence information was obtained from GenBank. *Acc1*, acetyl CoA carboxylase 1; *Adipoq*, adiponectin; *Adipor1*, adiponectin receptor 1; *Adipor2*, adiponectin receptor 2; *Atgl*, adipose triglyceride lipase; *β-actin*, actin beta; *Cd36*, fatty acid translocase, homologue of CD36; *Cpt1α*, carnitine palmitoyltransferase 1 alpha; *Cpt1β*, carnitine palmitoyltransferase 1 beta; *Dgat1*, diacylglycerol acyltransferase 1; *DsbA-L*, disulfide-bond-A oxidoreductase-like protein; *Ero1-Lα*, endoplasmic reticulum oxidoreductin 1-like protein alpha; *Fas*, fatty acid synthase; *Gpat*, glycerol-3-phosphate acyltransferase; *Had*, hydroxyacyl-CoA dehydrogenase; *Hprt*, hypoxanthine guanine phosphoribosyl transferase; *Hsl*, hormone-sensitive lipase; *Pparα*, peroxisome proliferator-activated receptor alpha; *Ppia*, peptidylprolyl isomerase A; *Tfrc*, transferrin receptor.

### Western blot analyses

p-AMPK in soleus muscles of the offspring was determined as previously described^18^. For the quantification of total T-cadherin levels in gastrocnemius muscles, the same procedure described in^18^ was used, with some modifications. Briefly, after blocking, the membranes were incubated with the primary antibody for T-cadherin rabbit anti-CDH13 (Sigma, Madrid, Spain), diluted 1/1000, and then with the goat anti-rabbit secondary antibody (LI-COR, USA), diluted 1/10000.

### Total adiponectin levels in adipose tissues

EWAT and inguinal white adipose tissue (IWAT) samples (200 mg) were homogenized in 400 μL of a buffer solution containing 100 mM Tris-HCl (SIGMA, Saint Louis, Missouri, USA), 250 mM sucrose (SIGMA, Saint Louis, Missouri, USA), 1 mM Pefabloc (SIGMA, Saint Louis, Missouri, USA) and a phosphatase inhibitor cocktail (SIGMA, Saint Louis, Missouri, USA) diluted 1/100. The homogenate was incubated for 30 minutes at 4 °C with agitation and then centrifuged at 1000 × g for 10 minutes at 4 °C to remove the lipids. Total adiponectin levels in the homogenate were measured using the rat adiponectin ELISA kit (Millipore, Barcelona, Spain) and normalized for protein content, which was determined using the Bradford protein assay^31^.

### Lipid content in mammary glands

Lipids were extracted from the mammary glands (100 mg) and quantified as previously described^9^.

### Statistical analysis

Data are expressed as the mean ± SEM. Differences among the groups were analyzed using repeated measures ANOVA, two-way ANOVA or Student’s t test. The specific statistical analyses used are noted in the figure legends and table footnotes. Statistical analyses were performed with SPSS (SPSS, Inc., Chicago, IL), and the level of statistical significance was set at bilateral 5%.

## Results

### GSPE supplementation increased the circulating levels of NEFAs and total adiponectin in dams

GSPE dams showed decreased liver weight (p = 0.032, Student’s t test) and a clear trend toward lower body weight gain compared with the VEH animals (p = 0.061, Student’s t test), which could be attributed to a decreased daily energy intake (p = 0.013, Student’s t test) (Table 2). Furthermore, the circulating levels of NEFAs were significantly higher in GSPE rats than in their counterparts (p = 0.015, Student’s t test), and this was translated to a lower R-QUICKI (p = 0.026, Student’s t test), which could suggest a decrease in insulin sensitivity (Table 2). GSPE treatment during lactation also produced a significant increase in the circulating levels of total adiponectin (p < 0.001, Student’s t test) (Table 2). Nevertheless, no significant changes between groups were observed for the plasma HMW-adiponectin, which is the more biologically active form of this adipokine^32^, and when total circulating levels of adiponectin were divided by the sum of all the white fat depots collected, which yields the effective production of adiponectin by white adipocytes^33^ (Table 2).

**Table 2:** Biometric, plasma parameters and energy intake of dams fed with chow and supplemented with vehicle or GSPE during lactation.

	VEH	GSPE
**Biometric parameters**		
Initial body weight (g)	278 ± 3	280 ± 4
Final body weight (g)	286 ± 3	280 ± 3
Body weight gain (g)	8 ± 4	−1 ± 2
RWAT (g)	3.15 ± 0.41	3.49 ± 0.29
MWAT (g)	1.87 ± 0.14	2.19 ± 0.21
OWAT (g)	4.93 ± 0.64	6.02 ± 0.69
Adiposity index (%)	3.81 ± 0.45	4.57 ± 0.42
Mammary gland (g)	11.0 ± 0.3	10.8 ± 0.5
Liver (g)	10.4 ± 0.1	9.8 ± 0.2*
Lean mass (g)	247 ± 3	240 ± 3
Lean mass (%)	86.3 ± 0.5	86.0 ± 0.9
Fat mass (g)	21.6 ± 2.1	24.3 ± 2.7
Fat mass (%)	7.54 ± 0.73	8.64 ± 0.92
**Plasma parameters**		
Glucose (mmol/L)	9.88 ± 0.24	10.1 ± 0.2
Insulin (ng/mL)	1.76 ± 0.27	1.83 ± 0.17
NEFAs (mmol/L)	0.22 ± 0.02	0.34 ± 0.04*
HOMA-IR	19.4 ± 3.4	20.7 ± 2.3
R-QUICKI	0.314 ± 0.006	0.294 ± 0.005*
Total adiponectin (μg/mL)	17.6 ± 0.7	21.8 ± 0.6*
HMW-adiponectin (μg/mL)	2.99 ± 0.55	4.41 ± 1.57
Total adiponectin/g WAT	1.72 ± 0.17	2.10 ± 0.19
Triglycerides (mmol/L)	0.37 ± 0.02	0.36 ± 0.04
Phospholipids (mmol/L)	2.11 ± 0.07	2.13 ± 0.07
Total cholesterol (mmol/L)	2.21 ± 0.11	2.14 ± 0.09
**Energy intake (kcal/day)**	**155 ± 2**	144 ± 3*

The ratio of the total adiponectin/g WAT was calculated by dividing the circulating levels of total adiponectin by the sum of the WAT depot weights (OWAT, MWAT and RWAT). Data are the mean ± SEM (n = 9). *Effect of GSPE treatment (Student’s *t* test, p < 0.05). MWAT, mesenteric white adipose tissue.

### GSPE intake during lactation enhanced lipid oxidation in dams

**Figure 1:**
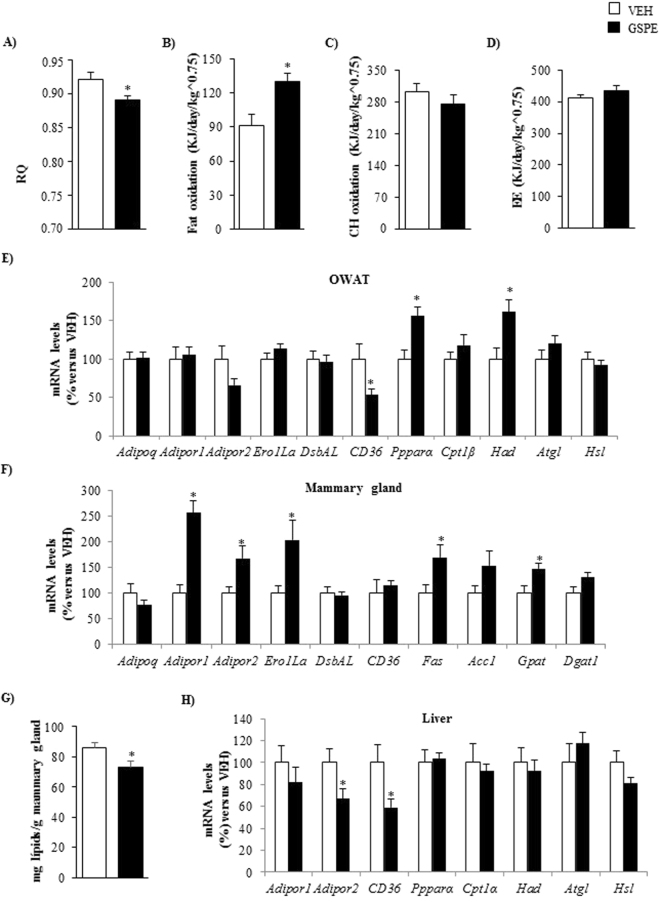
Respiratory quotient (RQ) (**A**), fat oxidation (**B**) carbohydrate oxidation (**C**) and energy expenditure (EE) (**D**) of dams that were fed with STD and supplemented with either GSPE (100 mg.kg^−1^.day^−1^) (GSPE group) or vehicle (VEH group) during lactation. For indirect calorimetric measurements, at day 19 of lactation, the rats received the corresponding treatment at 09:00 am. Four hours after (at 01.00 pm) the nursing rats were separated from their pups and were transferred from their cages to an acrylic box with free access to water but no access to food. After an initial acclimatisation period of 1 hour, the indirect calorimetric analyses were carried out for 3 hours (from 02:00 pm to 5:00 pm). The mRNA levels of genes involved in adiponectin metabolism in OWAT (**E**), mammary gland (**F**) and liver (**H**) as well as the concentration of lipids in the mammary gland (**G**) are also depicted. Data are the mean ± SEM (n = 8–9). *Effect of GSPE treatment (Student’s t test, p < 0.05). *Acc1*, acetyl CoA carboxylase 1; *Adipoq*, adiponectin; *Adipor1*, adiponectin receptor 1; *Adipor2*, adiponectin receptor 2; *Atgl*, adipose triglyceride lipase; *Cd36*, fatty acid translocase, homologue of CD36; *Cpt1α*, carnitine palmitoyltransferase 1 alpha; *Dgat1*, diacylglycerol O-acyltransferase 1; *DsbA-L*, disulfide-bond-A oxidoreductase-like protein; *Ero1-Lα*, endoplasmic reticulum oxidoreductin 1-like protein alpha; *Fas*, fatty acid synthase; *Gpat*, glycerol-3-phosphate acyltransferase; *Had*, hydroxyacyl-CoA dehydrogenase; *Hsl*, hormone-sensitive lipase; *Pparα*, peroxisome proliferator-activated receptor alpha.

GSPE dams exhibited a significantly lower RQ than VEH dams (p = 0.025, Student’s t test) (Fig. 1A) and, consequently, showed a shift toward higher lipid oxidation (p = 0.006, Student’s t test) (Fig. 1B), indicating that GSPE intake during lactation enhanced the use of fat instead of carbohydrates as an energy source.

### GSPE-treated dams showed changes in the expression of genes involved in lipid metabolism and adiponectin signaling

In ovaric white adipose tissue (OWAT), the supplementation of GSPE during lactation significantly increased the mRNA levels of the gene encoding the master regulator of β-oxidation, peroxisome proliferator-activated receptor alpha (PPARα) (p = 0.006, Student’s t test) (Fig. 1E). A very similar pattern of expression was observed for the β-oxidation-related gene hydroxyacyl-CoA dehydrogenase (*Had*)^18^ (p = 0.015, Student’s t test) (Fig. 1E). In OWAT, GSPE dams also displayed a significant drop in the gene encoding the fatty acid transporter CD36 (p = 0.046, Student’s t test) and showed a clear trend towards decreased mRNA levels of the adiponectin receptor 2 (*Adipor2*) (p = 0.086, Student’s t test) (Fig. 1E).

Adiponectin receptor 1 (*Adipor1*) mRNA levels were sharply over-expressed in the mammary gland of GSPE rats (p < 0.001, Student’s t test) (Fig. 1F). This behavior was also observed, although to a lesser extent, for *Adipor2* (p = 0.023, Student’s t test) and the gene encoding the endoplasmic reticulum oxidoreductin 1-like protein alpha (Ero1Lα), a key enzyme involved in HMW adiponectin secretion^34^ (p = 0.023, Student’s t test) (Fig. 1F). GSPE-treated dams also displayed a significant increase in the mRNA levels of the key lipogenic genes including fatty acid synthase (*Fas*) (p = 0.038, Student’s t test) and glycerol-3-phosphate acyltransferase (*Gpat*) (p = 0.017, Student’s t test) and residually elevated gene expression levels of diacylglycerol O-acyltransferase 1 (*Dgat1*) (p = 0.063, Student’s t test) in this tissue (Fig. 1F). These animals also showed a significant decrease in the lipid content of the mammary gland (p = 0.041, Student’s t test) (Fig. 1G).

The administration of GSPE during lactation also produced a significant decrease in the hepatic mRNA levels of *Adipor2* and *CD36* genes (p = 0.050 and p = 0.035, respectively, Student’s t test) (Fig. 1H).

### CAF intake induced MetS in the offspring

**Figure 2:**
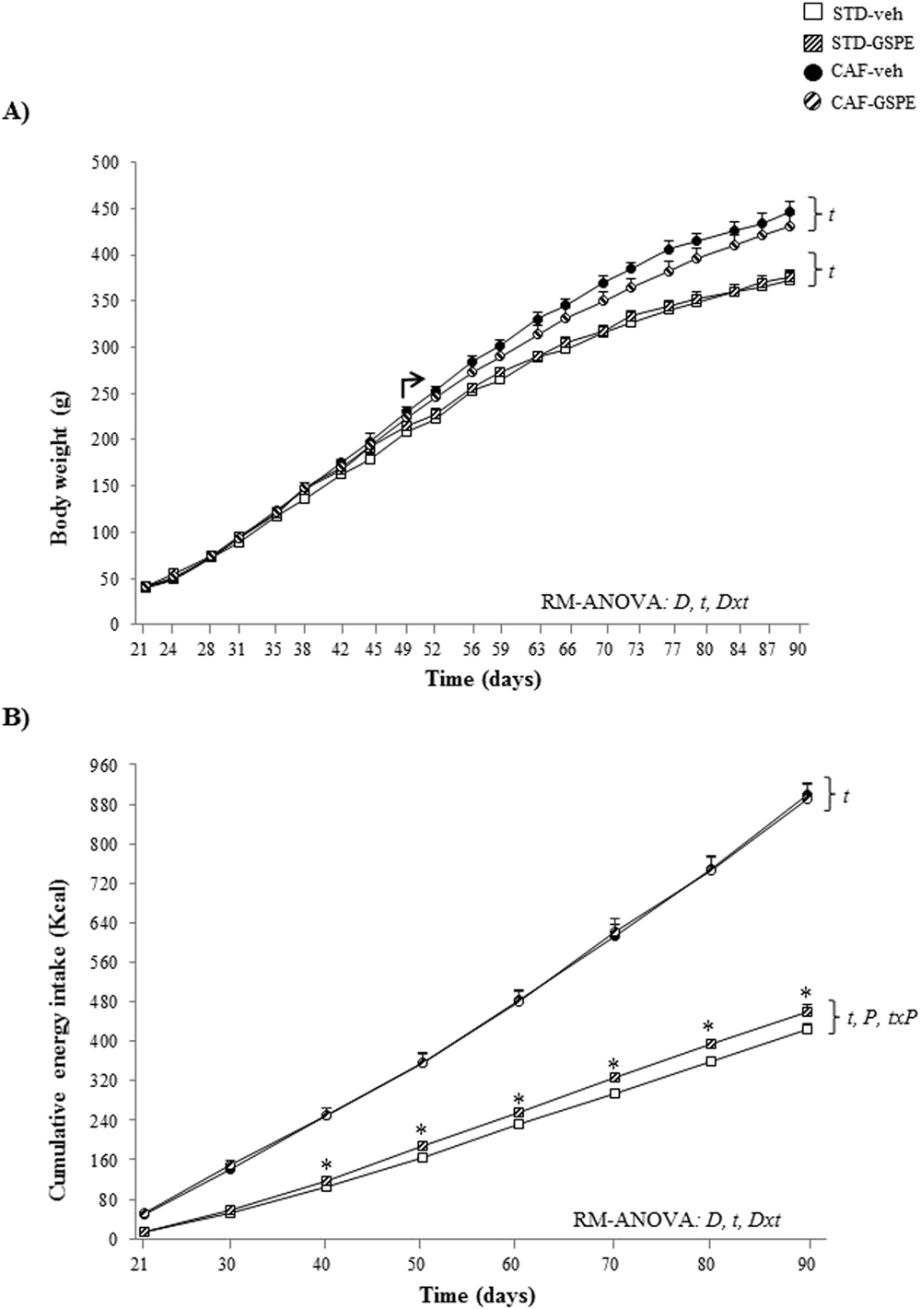
The evolution of body weight (**A**) and the cumulative energy intake (**B**) in male offspring of rats supplemented with GSPE or vehicle during lactation. Body weight was recorded twice weekly and energy intake was documented every 10 days. Both parameters were analysed by a repeated measures (RM-) ANOVA with time as a within-subject factor and diet (STD or CAF) and metabolic programming effect (vehicle or GSPE) as between-subject factors. When the interaction between diet and time was statistically significant according to RM-ANOVA, another RM-ANOVA was also used to analyse the effects of time and metabolic programming within the STD and the CAF groups. When a significant interaction between metabolic programming effect and time was found within the STD groups under the RM-ANOVA, Student’s t test was used to compute pairwise comparisons between these groups. Data are the mean ± SEM (n = 10–13). *t*: effect of time; D: effect of the type of diet; *D*x*t*: the interaction between diet and time; *P*: metabolic programming effect of GSPE; *txP*: the interaction between time and metabolic programming effect of GSPE (p < 0.05, RM-ANOVA). The arrow indicates the day from which significant differences in body weight between the CAF and STD groups were found, i.e., the effect of diet type (two-way ANOVA, p < 0.05). *Metabolic programming effect of GSPE within STD groups (Student’s *t* test, p < 0.05).

**Figure 3:**
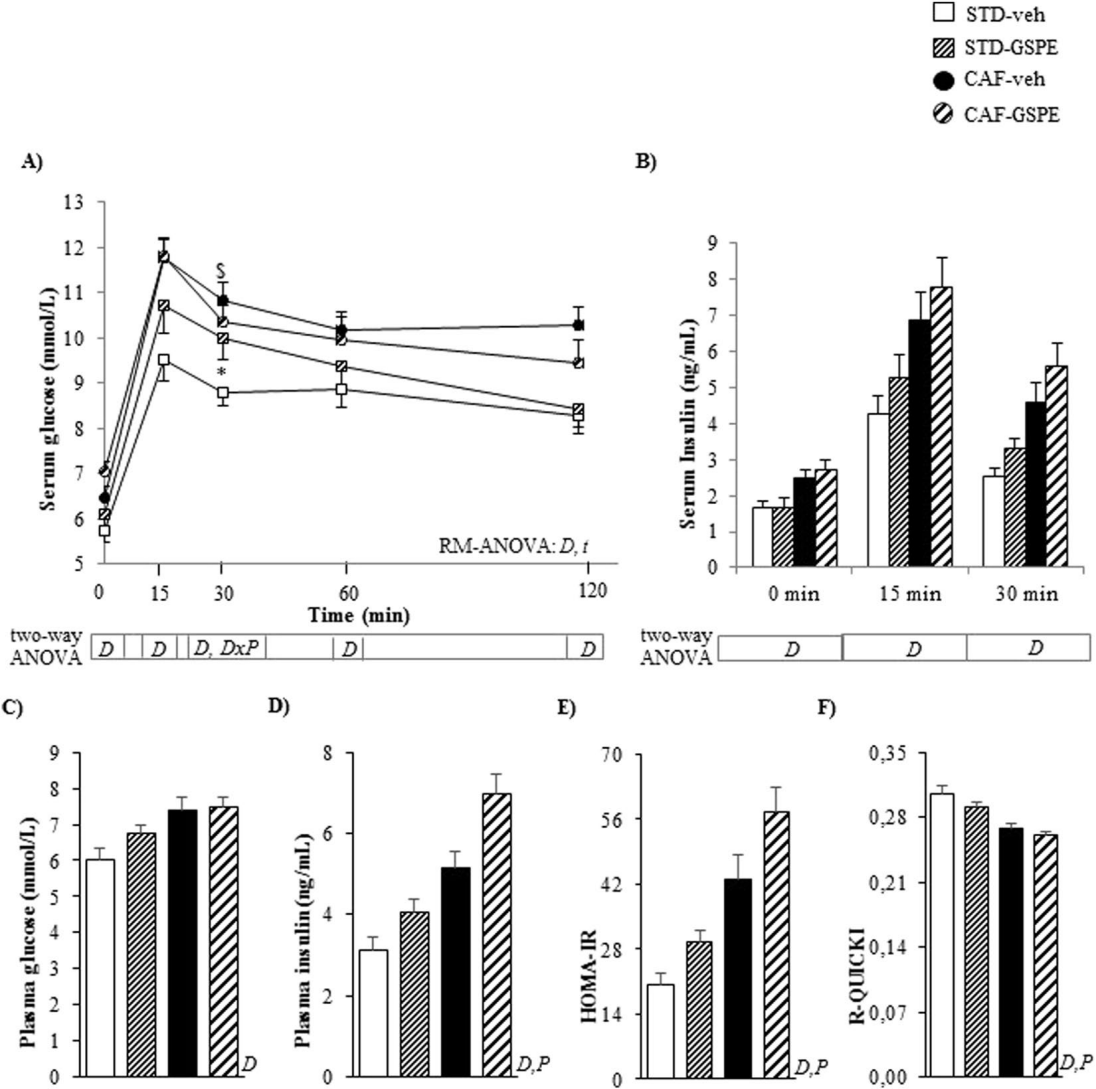
Circulating levels of glucose (**A**) and insulin (**B**) after an OGTT (2 g of glucose kg^−1^ of body weight) performed on postnatal day 80 in male offspring of rats supplemented with GSPE or vehicle during lactation. The plasma levels of glucose (**C**) and insulin (**D**), as well the HOMA-IR (**E**) and the R-QUICKI (**F**) indexes obtained at the end point are also depicted. Data are the mean ± SEM (n = 10–13). The evolution of the circulating levels of glucose during the OGTT was analysed by a repeated measures (RM-) ANOVA with time as a within-subject factor and diet (STD or CAF) and metabolic programming effect (vehicle or GSPE) as between-subject factors. At each study point, differences among groups for these parameters were assessed using a two-way ANOVA analysis (2 × 2 factorial designs: diet (STD or CAF) × metabolic programming effect (vehicle or GSPE)). When the interaction between diet and metabolic programming effect was statistically significant according to two-way ANOVA, Student’s t test was used to compute pairwise comparisons between groups (i.e., the effect of metabolic programming effect within diet groups and the effect of diet within VEH and GSPE groups). Two-way ANOVA was used to evaluate differences in the circulating levels of insulin at each point of the OGTT and in the serum levels of glucose, insulin, HOMA-IR and R-QUICKI at the end point. *D*: effect of the type of diet; *t*: effect of time; *P*: metabolic programming effect of GSPE (repeated measures ANOVA or two-way ANOVA, p < 0.05). ^$^The effect of diet within vehicle groups; ^*^metabolic programming effect of GSPE within STD groups (Student’s *t* test, p < 0.05).

**Figure 4:**
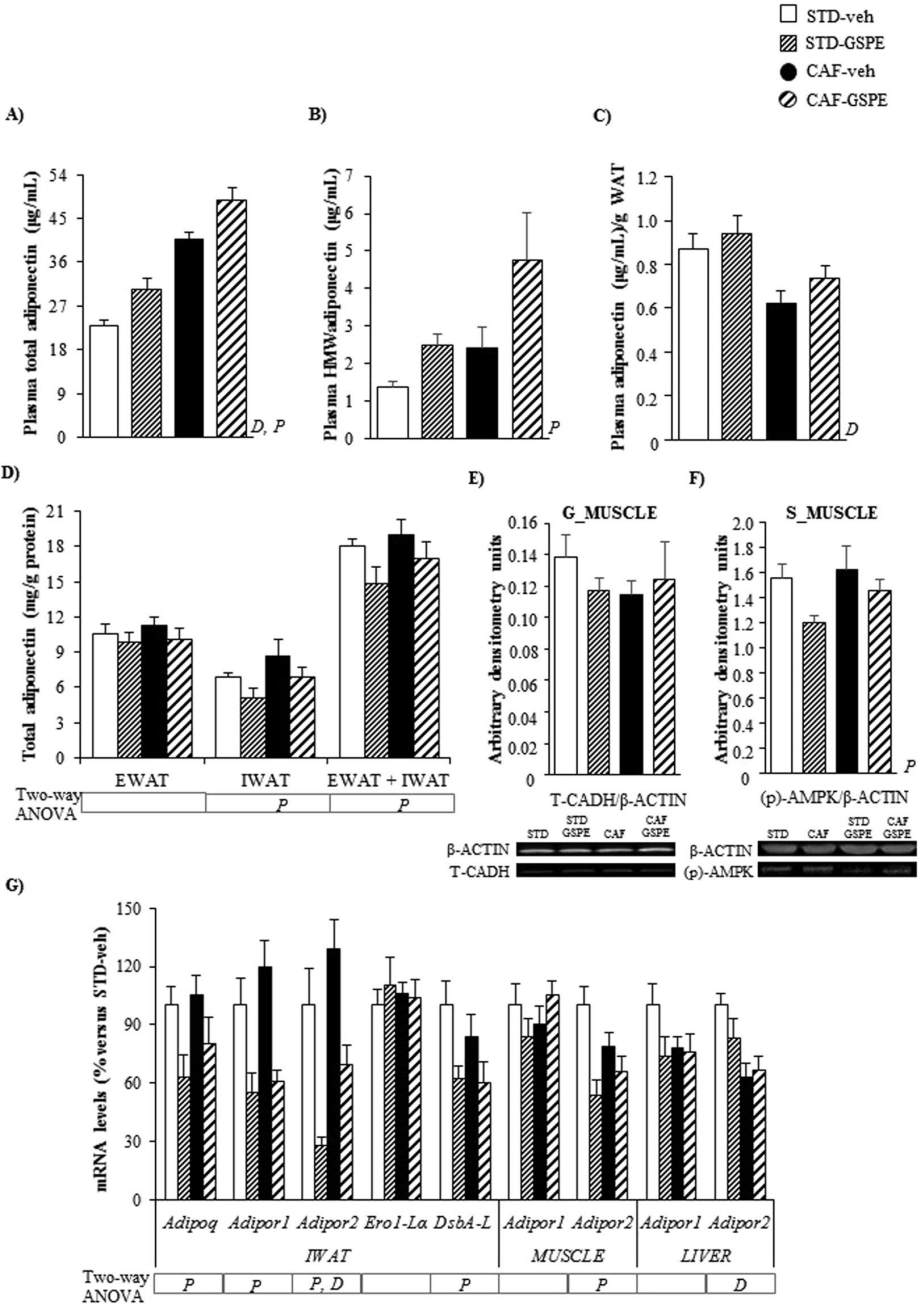
Plasma levels of total adiponectin (**A**), HMW-adiponectin (**B**), plasma adiponectin/WAT weight ratio (**C**) and adiponectin content in EWAT and IWAT depots (**D**) in male offspring of rats supplemented with GSPE or vehicle during lactation. The mRNA levels of genes involved in adiponectin metabolism in IWAT, soleus muscle and liver (**G**) as well as the protein levels of T-cadherin in gastrocnemius (**G**) muscle (**E**) and p-AMPK in soleus (S) muscle (**F**) are also depicted. The ratio of total adiponectin/g WAT was calculated by dividing the circulating levels of total adiponectin by the sum of the WAT depot weights (EWAT, mesenteric white adipose tissue –MWAT-, RWAT and IWAT). Data are the mean ± SEM (n = 10–13) for the total plasma adiponectin, adiponectin/WAT weight ratio and adiponectin content in white adipose tissue depots and the mean ± SEM (n = 8) for the other analyses. Two-way ANOVA analysis (2 × 2 factorial designs: diet (STD or CAF) × metabolic programming effect (vehicle or GSPE) was used to evaluate differences in these parameters. *D*: effect of the type of diet; *P*: metabolic programming effect of GSPE (two-way ANOVA, p < 0.05). *Adipoq*, adiponectin; *Adipor1*, adiponectin receptor 1; *Adipor2*, adiponectin receptor 2; *DsbA-L*, disulfide-bond-A oxidoreductase-like protein; *Ero1-Lα*, endoplasmic reticulum oxidoreductin 1-like protein alpha; EWAT: epididymal white adipose tissue; HMW-adiponectin: high-molecular weight adiponectin; IWAT: inguinal white adipose tissue; p-AMPK, phosphorylated AMP-activated protein kinase; T-CADH: T-cadherin.

Regardless of the treatment received, the CAF-fed groups displayed higher body weight than their lean counterparts from day 49 of life onwards (repeated measures ANOVA, p < 0.05) (Fig. 2A). This effect on body weight could be partially explained by the increased cumulative energy intake observed in these animals during the experimental period (repeated measures ANOVA, p < 0.001) (Fig. 2B). As expected^25^, both CAF-fed groups showed increased final body weight and developed MetS-like alterations, including obesity, dyslipidemia, glucose intolerance and insulin resistance (p < 0.05, two-way ANOVA for all the parameters analysed) (Table 3, Fig. 3). CAF consumption increased the circulating levels of total adiponectin in both CAF-veh and CAF-GSPE offspring (p < 0.001, two-way ANOVA) (Fig. 4A). Moreover, a similar trend was observed for HMW-adiponectin, although the differences were not statistically significant (p = 0.059, two-way ANOVA) (Fig. 4B). However, when the circulating levels of total adiponectin were normalized to the total WAT weight, a significant decrease in this ratio was observed in both CAF-fed groups compared with STD groups (p = 0.001, two-way ANOVA) (Fig. 4C).

**Table 3:** Biometric and plasma parameters in 90-day-old male offspring of rats supplemented with GSPE or vehicle during lactation.

	STD-veh	STD-GSPE	CAF-veh	CAF-GSPE	Two-way ANOVA
**Biometric parameters**					
Initial body weight (g)	40 ± 2	41 ± 1	41 ± 1	42 ± 1	—
Final body weight (g)	373 ± 9	375 ± 8	446 ± 12	431 ± 13	*D*
Body weight gain (g)	333 ± 8	334 ± 7	405 ± 12	389 ± 12	*D*
Liver (g)	12.0 ± 0.4	11.6 ± 0.4	14.3 ± 0.5	13.8 ± 0.5	*D*
RWAT (g)	6.47 ± 0.45	8.33 ± 0.84	18.6 ± 1.7	17.7 ± 1.0	*D*
MWAT (g)	4.28 ± 0.27	5.00 ± 0.40	8.46 ± 0.57	9.03 ± 0.67	*D*
EWAT (g)	5.67 ± 0.48	7.29 ± 0.60	14.9 ± 1.3	15.2 ± 1.3	*D*
IWAT (g)	11.9 ± 1.0	13.0 ± 1.1	29.3 ± 3.0	28.00 ± 2.6	*D*
Adiposity index (%)	7.75 ± 0.54	9.02 ± 0.66	16.1 ± 1.0	16.3 ± 0.9	*D*
Gastrocnemius muscle (g)	1.74 ± 0.05	1.78 ± 0.06	1.86 ± 0.02	1.81 ± 0.04	—
Soleus muscle (g)	0.147 ± 0.006	0.147 ± 0.006	0.142 ± 0.04	0.143 ± 0.04	—
Lean mass (g)	322 ± 9	319 ± 8	332 ± 5	321 ± 8	—
Lean mass (%)	86.2 ± 0.8	85.0 ± 1.1	74.8 ± 1.4	74.6 ± 1.3	*D*
Fat mass (g)	32.0 ± 2.7	36.9 ± 4.2	93.1 ± 8.4	92.1 ± 7.1	*D*
Fat mass (%)	8.6 ± 0.8	9.8 ± 1.1	20.5 ± 1.4	21.1 ± 1.3	*D*
**Plasma parameters**					
Total cholesterol (mmol/L)	1.65 ± 0.11	1.90 ± 0.13	2.20 ± 0.09	2.21 ± 0.09	*D*
Triglycerides (mmol/L)	1.75 ± 0.32	1.27 ± 0.18	3.67 ± 0.39	3.93 ± 0.22	*D*
NEFAs (mmol/L)	0.28 ± 0.04	0.23 ± 0.02	0.34 ± 0.02	0.33 ± 0.03	*D*
Phospholipids (mmol/L)	1.77 ± 0.09	1.90 ± 0.04	2.38 ± 0.12	2.60 ± 0.09	*D*
MCP-1 (ng/mL)	8.64 ± 0.37	8.92 ± 0.37	12.0 ± 0.4	11.1 ± 0.8	*D*
Leptin (ng/mL)	9.33 ± 0.63	9.49 ± 0.87	39.6 ± 3.6	41.8 ± 2.8	*D*

Data are the mean ± SEM (n = 10–13). Two-way ANOVA analysis (2 × 2 factorial designs: diet (STD or CAF) × metabolic programming effect (vehicle or GSPE) was used to evaluate differences in these parameters. *D*: effect of the type of diet, (two-way ANOVA, p < 0.05). MWAT, mesenteric white adipose tissue.

### Maternal supplementation of GSPE during lactation increased energy intake in normoweight offspring

STD-GSPE animals showed higher cumulative caloric intake over time than the STD-veh group (repeated measures ANOVA, p = 0.003), being this effect statistically significant from day 40 of life onwards (p < 0.05, Student’s t test) (Fig. 2B). In addition, these animals also displayed a substantial enlargement of EWAT and retroperitoneal white adipose tissue –RWAT- (28.6% and 28.7% greater, respectively) in comparison with their counterparts, although the differences were not statistically significant (Table 3).

### GSPE administration to dams during lactation programmed the offspring for insulin resistance

The OGTT revealed that both STD-GSPE and CAF-GSPE animals displayed numerically higher serum glucose levels at baseline than their counterparts, although the differences were not statistically significant (p = 0.060, two-way ANOVA) (Fig. 3A). After the glucose load, STD-GSPE offspring attained significantly higher circulating levels of glucose (after 30 minutes) than the STD-veh rats (p = 0.045, Student’s t test) (Fig. 3A). Furthermore, both STD-GSPE and CAF-GSPE offspring showed a residual increase in insulin circulating levels 30 minutes after the glucose gavage (p = 0.058, two-way ANOVA) (Fig. 3B), and this effect was more evident in STD-GSPE animals (p = 0.057, Student’s t test versus STD-veh group) (Fig. 3B).

At the end point, both STD-GSPE and CAF-GSPE rats showed a significant rise in plasma insulin levels (p = 0.002, two-way ANOVA) (Fig. 3D). This change resulted in a higher degree of insulin resistance (p = 0.002, two-way ANOVA) and lower insulin sensitivity (p = 0.042, two-way ANOVA), measured as the HOMA-IR and R-QUICKI indexes, respectively, in both STD-GSPE and CAF-GSPE animals than in their counterparts (Fig. 3E and F).

### The offspring of GSPE-treated dams displayed increased circulating levels of total and HMW adiponectin

The administration of GSPE to dams during lactation significantly increased the plasma levels of total adiponectin in both STD-fed and CAF-fed offspring (p < 0.001, two-way ANOVA) (Fig. 4A). This effect was also observed for the circulating levels of HMW-adiponectin, but to a lesser extent (p = 0.025, two-way ANOVA) (Fig. 4B). No metabolic programming effects of GSPE were found when the total plasma levels of this adipokine were adjusted per total WAT weight (Fig. 4C).

### Maternal intake of GSPE during lactation produced clear programming effects related to adiponectin signaling in IWAT, muscle and liver

Overall, STD-GSPE and CAF-GSPE offspring showed significantly lower adiponectin content in the IWAT than their counterparts (p = 0.023, two-way ANOVA). This pattern was maintained when the adiponectin content was expressed as the sum of the concentration obtained in both EWAT and IWAT (p = 0.050, two-way ANOVA) (Fig. 4D).

GSPE produced profound metabolic programming effects at the mRNA level in the IWAT of both STD-GSPE and CAF-GSPE groups, significantly decreasing the expression of the genes encoding adiponectin (p = 0.012, two-way ANOVA), adiponectin receptors 1 and 2 (p < 0.001, two-way ANOVA) and disulfide-bond-A oxidoreductase-like protein (DsbA-L) (p = 0.006, two-way ANOVA) (Fig. 4G). A*dipor2* mRNA was also downregulated in the soleus muscle of both animal groups (p = 0.001, two-way ANOVA) (Fig. 4G).

GSPE treatment of dams during lactation significantly decreased p-AMPK levels in the soleus muscle of their both normoweight and obese offspring (p = 0.047, two-way ANOVA), especially in STD-fed animals (Fig. 4F).

### GSPE supplementation in lactating dams enhanced EE and locomotor activity in the offspring

**Figure 5:**
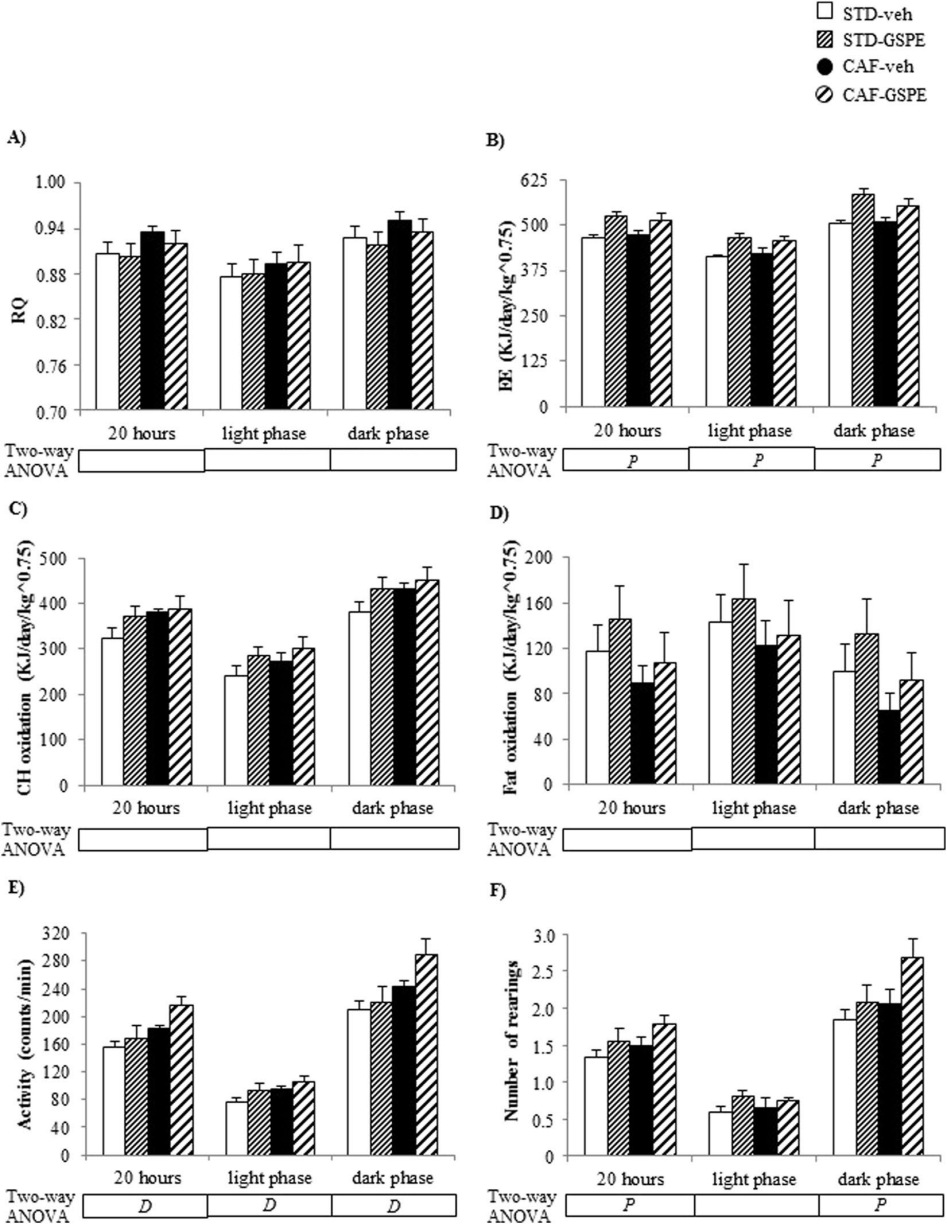
Respiratory quotient (RQ) (**A**), energy expenditure (EE) (**B**), carbohydrate oxidation (**C**), fat oxidation (**D**), spontaneous locomotor activity (**E**) and number of rearings (**F**) in male offspring of rats supplemented with GSPE or vehicle during lactation. Indirect calorimetric measurements were performed at day 83–84 of age with free access to water, in *ad libitum* conditions and during 20 hours (from 12.00 pm to 08.00 am) after allowed to become acclimated to the acrylic cages for 3.5 hours (from 08:30 am to 12:00 pm). Data are the mean ± SEM (n = 10–13). Two-way ANOVA analysis (2 × 2 factorial designs: diet (STD or CAF) × metabolic programming effect (vehicle or GSPE) was used to evaluate differences in these parameters. *D*: effect of the type of diet; *P*: metabolic programming effect of GSPE (two-way ANOVA, p < 0.05).

GSPE administration to dams during lactation significantly increased EE during both the light and the dark phases in both the STD-GSPE and CAF-GSPE groups (p < 0.001, two-way ANOVA) (Fig. 5B). Furthermore, both STD-GSPE and CAF-GSPE offspring displayed a significant greater number of rearings than their counterparts when all the data collected during 20 hours were considered (p = 0.046, two-way ANOVA) and during the dark phase (p = 0.050, two-way ANOVA) (Fig. 5F). These animals also showed overall numerically higher spontaneous locomotor activity than their counterparts, although the differences were not statistically significant (p = 0.058, two-way ANOVA) (Fig. 5E).

## Discussion

In this study, we reported that both normoweight and CAF-fed obese young adult male offspring of nursing rats supplemented with moderate doses of GSPE during lactation showed increased circulating levels of total and HMW-adiponectin. The ability of some polyphenols, such as oleuropein^35^ or green tea polyphenols^36^, to elevate the total blood adiponectin levels has been previously reported in rats fed obesogenic diets. Moreover, Mukai *et al*.^15^ demonstrated that the offspring of dams that received a low-protein diet containing adzuki bean polyphenols during gestation displayed significantly increased total adiponectin plasma levels in adulthood. However, to the best of our knowledge, this is the first study describing a metabolic programming effect of polyphenols on both total and HMW-adiponectin when these bioactive compounds are administered during lactation. The rise in plasma adiponectin observed in the offspring of GSPE-treated dams contrasted with the lower adiponectin content observed in WAT. The down-regulation of the IWAT mRNA levels of the genes encoding adiponectin and DsbA-L, a key regulator of adiponectin multimerization and secretion^37^, and the unchanged effective adipocyte production of adiponectin estimated as the adiponectin/WAT ratio^33^, suggest decreased adiponectin production by adipose tissue. To assess whether increased levels of plasma adiponectin were due to lowered clearance, we determined the muscular levels of the adiponectin-binding protein T-cadherin, which is mainly expressed in the muscle, heart and aorta and plays a crucial role in the regulation of the circulating levels of adiponectin^38,39^. Nevertheless, GSPE supplementation during lactation was not associated with altered T-cadherin content in the gastrocnemius muscle of these animals (Fig. 3E). Further studies focused on the quantification of this protein in other tissues, on the analyses of other molecular forms of adiponectin or on other mechanisms that could also account for the circulating levels of this adipocytokine, such as hepatic clearance from the bloodstream^40^, are needed to clarify this issue.

Adiponectin, especially in its HMW form, plays a key role in glucose and lipid metabolism. It binds to its receptors AdipoR1 and AdipoR2 and thus acts as an insulin sensitizer by enhancing lipid oxidation and inhibiting lipogenesis and gluconeogenesis in liver and promoting glucose uptake and fatty oxidation in skeletal muscle^32^. However, we observed an increase in both total and HMW-adiponectin in both STD-GSPE and CAF-GSPE animals, which was accompanied by hyperinsulinemia and increased insulin resistance and decreased insulin sensitivity. Considering the key role of adiponectin receptors in the actions of this adipocytokine on insulin sensitivity in muscle^32^, liver^32^ and adipose tissue^41,42^, these effects can be partly attributed to the decreased gene expression of these receptors observed in IWAT (*Adipor1* and *Adipor2*) and soleus muscle (*Adipor2*) of both normoweight and obese offspring of GSPE-supplemented dams. The reduction in soleus muscle and adipose tissue *Adipor1* and *Adipor2* mRNA levels observed in the insulin-resistant ob/ob mice^43^ and the fact that the amelioration of insulin resistance by exercise training was accompanied by increased *ADIPOR1* and *ADIPOR2* gene expression in human subcutaneous fat^41^ further corroborates our hypothesis. These results, together with the down-regulation of p-AMPK, a downstream post-receptor target of adiponectin that enhances glucose uptake and fatty acid oxidation^32^, observed in the soleus muscle of STD-GSPE and CAF-GSPE rats, clearly suggest a development of an adiponectin-resistance like phenotype in these animals due to maternal intake of GSPE during lactation. Further research focused on the analyses of the mRNA and/or protein levels of the adiponectin receptors and different downstream post-receptor targets of adiponectin in other muscles and white adipose tissues would be of great value to confirm the development of adiponectin resistance in the offspring of nursing rats treated with GSPE. Remarkably, the deleterious metabolic programming effects of GSPE on adiponectin signaling and insulin sensitivity seem more evident in STD-GSPE than in CAF-GSPE animals because, when compared with their respective counterparts, the normoweight rats displayed a sharper decrease of phosphorylated AMPK levels and *AdipoR2* gene expression in soleus muscle and showed decreased glucose clearance 30 minutes after the glucose load. This could be tentatively attributed to the numerically higher EWAT and RWAT weights observed in STD-GSPE animals in comparison with STD-veh rats, which could negatively impact on insulin sensitivity, since different studies carried out in rodents have shown that the specific surgical removal of visceral fat (EWAT, RWAT and/or perinephric fat) ameliorated insulin resistance or diabetes mellitus^44–46^.

Different scientific evidences indicate that adiponectin resistance is involved in skeletal muscle wasting^47–49^. This deleterious effect is mediated, at least in part, by a down-regulation of AdipoR1, since the mRNA and protein expression levels of this receptor significantly decreased in the skeletal muscle of patients with hyperadiponectinemia and chronic heart failure^48^, a disease that is associated with skeletal muscle adiponectin resistance, muscle atrophy, fiber type shift and impaired skeletal muscle metabolism^47,48^. In mice, specific suppression of AdipoR1 in muscle resulted in a decreased mitochondrial content and activity, and in a reduction of the number of oxidative type I fibers in skeletal muscle^49^. In our study, the lack of down-regulation of the *AdipoR1* mRNA levels observed in the soleus of both STD-GSPE and CAF-GSPE offspring would contribute to explain, at least in part, that the adiponectin resistance-like phenotype observed in these animals was not also manifested with a decreased weight of this tissue. Furthermore, no changes were found either in the gastrocnemius weight or in lean mass content measured by quantitative magnetic resonance, which provides an accurate measurement of muscle mass^50^. Therefore, it seems unlikely that GSPE produced this detrimental metabolic programming effect, although the dissection of other specific muscles, such as extensor digitorum longus, anterior quadriceps and tibialis anterior, and additional analyses related with muscle strength, fiber type and size and mitochondrial structure and activity would be useful to corroborate this hypothesis.

Previously, we demonstrated that the normoweight adult offspring of dams supplemented with GSPE during pregnancy and lactation displayed enhanced whole-body fat oxidation but did not show changes in EE^18^. Intriguingly, in this study, maternal intake of GSPE during lactation did not affect lipid oxidation but increased EE in both normoweight and CAF-fed obese offspring. The discrepancies between both studies may rely on either the dose of polyphenols used (25 vs 100 mg.kg^−1^.day^−1^), the length and the period of which the treatment was performed (pregnancy and lactation vs lactation) or the different age of the animals (170 vs 90 days). This increase of EE can be attributed, at least in part, to the increased locomotor activity observed in both STD-GSPE and CAF-GSPE rats. Furthermore, in STD-GSPE offspring this effect could be understood as a compensatory mechanism addressed to counteract the significantly higher energy intake and the clear trend toward increased EWAT and RWAT weights observed in these animals when compared with their counterparts. Additional research is needed to elucidate whether the enhancement of EE observed in both STD-GSPE and CAF-GSPE animals would be also related with other mechanisms, such as an activation of thermogenesis in brown adipose tissue (BAT) and/or an improvement of mitochondrial function in muscle and BAT, as was previously described in mice that were supplemented with resveratrol^51^. Furthermore, the effects of adiponectin signaling on the modulation of EE cannot be discarded, although evidences from different studies indicates a controversial role of this adipocytokine on this parameter, suggesting both stimulatory^52^ and inhibitory effects^53^.

Various lines of preclinical evidence suggest that changes in milk composition can increase the risk of the offspring suffering metabolic disturbances, such as insulin resistance, obesity and inflammation^54–56^. Therefore, the effects described in this study might be explained by changes in milk composition. Since it has been suggested that polyphenols present in food by-products, such as grape pomace, can be transferred to the milk^57^, direct signaling of grape seed polyphenols through maternal milk might also contribute, at least in part, to the metabolic programming effect observed in the offspring. Another possibility is related to changes in the endogenous components of milk. It has been shown that maternal adiponectin is passed through milk to lactating offspring^58^. Moreover, in humans, increased adiponectin content of maternal milk is associated with a higher risk of obesity in 2-year-old children, an effect that can be attributed to the central effects of adiponectin in the hypothalamus modulating food intake^59^. We found that maternal GSPE intake was associated with significant increases in plasma adiponectin in mothers and food intake in their STD offspring, which also displayed a substantial, but not significant, accretion of RWAT and EWAT depots. These findings are consistent with the effects of increased milk adiponectin reported by Brunner *et al*.^59^. Therefore, it is plausible to hypothesize that the increase in circulating adiponectin found in dams treated with GSPE resulted in higher levels of this adipokine in milk. In turn, this alteration could have driven the metabolic programming in the offspring. Moreover, it could be speculated that early exposure to higher levels of adiponectin was the main cause of adiponectin resistance observed in the offspring of GSPE-treated dams. However, the hormonal content of milk is complex and changes in other hormones or signaling molecules such as ghrelin, leptin, insulin or TNFα, among others, could have conditioned the neuro-endocrine system of the offspring during the lactation period^58^. Finally, another plausible explanation can be found in the reduced transfer of essential fatty acids or in the increased supply of saturated fatty acids to the pups^54–56^. Jin and collaborators showed that transgenic mice overexpressing adiponectin displayed increased lipid accumulation in the mammary gland, which resulted in excessive long-chain saturated fatty acids in milk, whereas adiponectin-KO mice showed enhanced production of inflammatory cytokines in this tissue. However, in both cases, an inflammatory response was triggered in the offspring^54^. These results strongly suggest that optimal levels of circulating adiponectin are needed to maintain a favorable mammary gland metabolism, a normal lipid supply to the milk and an optimal offspring’s health^54^. Intriguingly, in this study, despite GSPE-supplemented dams displaying increased circulating levels of total adiponectin, they showed decreased levels of total lipids in the mammary glands. Since fat levels in mammary glands roughly reflect the milk lipid content^54,55^, it can be hypothesized that the deleterious metabolic programming effects produced by GSPE in both normoweight and obese offspring are related to a lower lipid supply to the milk produced due to GSPE supplementation in dams during lactation. Hsieh and co-workers demonstrated that the development of insulin resistance in the offspring of mice lacking phosphoenolpyruvate carboxykinase in mammary gland adipocytes was due to a 40% reduction in the triglyceride content of milk during lactation^55^, which could support this hypothesis. Nevertheless, GSPE-treated dams displayed an over-expression of lipogenic genes (*Fas*, *Gpat* and, to a lesser extent, *Dgat1*) in their mammary glands, which would indicate increased lipid synthesis and, consequently, increased fat content in milk. In this scenario, the lower mammary gland lipid content observed in GSPE nursing rats could reflect a higher suckling capacity of their offspring, which would imply a higher lipid supply and energy intake in these animals than in their counterparts. The increased cumulative caloric intake and the slight adiposity accretion observed in STD-GSPE animals would agree with this hypothesis. In addition, the increased EE observed in both STD-GSPE and CAF-GSPE offspring could contribute to explain that these animals did not display greater body weight at weaning than the progeny of dams supplemented with the vehicle. In mice, dams overexpressing adiponectin produced more long chain saturated fatty acids in their milk and this resulted in inflammation and alopecia in their offspring^54^. In the present study, whether the deletereous effects on insulin sensitivity and adiponectin signaling observed in both STD-GSPE and CAF-GSPE offspring could be produced by an excessive content of saturated fatty acids in dam’s milk that were supplement with GSPE deserves further research. In rodents, between 10 and 20% of triglycerides that comprise the milk come from NEFAs derived from white adipose tissue and the circulation, whereas the remaining amount is generated by de novo synthesis in the mammary gland^60^. Thus, the increased circulating levels of NEFAs, the sharp increase in mammary gland *AdipoR1* and *AdipoR2* mRNA levels, and the down-regulation of *AdipoR2* gene expression in liver and OWAT observed in GSPE dams could be a mechanism for increasing the fatty acid supply to the mammary fat pad. Altogether, these results would indicate a metabolic adaptation to maximize NEFA availability by the mammary gland, which was also suggested by the decreased expression of the fatty acid transporter-related gene *CD36* observed in both OWAT and the liver of the GSPE-treated rats. GSPE dams also displayed increased whole-body lipid oxidation and an over-expression of the β-oxidation-related genes *Pparα* and *Had* in the OWAT. These results would suggest that GSPE supplementation in nursing rats could enhance fatty acid utilization in white adipose tissue, an effect that could be attributed, at least in part, to the high availability of NEFAs.

One limitation of the present study is the fact that we did not collect the milk of the nursing rats and, therefore, we cannot demonstrate a direct association between changes in dam’s milk composition as a consequence of GSPE supplementation and the observed effects of this polyphenol extract in their normoweight and obese offspring. New studies analyzing the milk composition of dams will be of great value to shed more light in the mechanisms involved in the aforementioned metabolic programming effects of GSPE.

In conclusion, we demonstrated that GSPE supplementation in lactating dams programmed their normoweight and obese offspring towards increased circulating total and HMW-adiponectin; however, it also induced an adiponectin-resistant-like phenotype. This phenotype was apparent in the following observations: 1) decreased gene expression of adiponectin receptors (*AdipoR1* and *AdipoR2* in IWAT and *AdipoR2* in soleus muscle); 2) hyperinsulinemia, decreased insulin sensitivity and increased insulin resistance; and 3) decreased levels of the downstream post-receptor target of adiponectin, p-AMPK, in the soleus muscle. Altogether, our results do not support the intake of GSPE at moderate doses during lactation as an effective strategy to improve the health of the offspring. Despite the numerous beneficial effects attributed to polyphenol consumption, including GSPE, in different stages of life, our findings raise concerns about the nutraceutical supplementation of GSPE during lactation, which is a critical stage that affects infant development and metabolism during their entire lives.
